# Removal of 10-nm contaminant particles from Si wafers using CO_2 _bullet particles

**DOI:** 10.1186/1556-276X-7-211

**Published:** 2012-04-11

**Authors:** Inho Kim, Kwangseok Hwang, JinWon Lee

**Affiliations:** 1 Department of Mechanical Engineering, Pohang University of Science and Technology (POSTECH), Hyoja 31, Pohang, Kyungbuk 790-784, South Korea; 2 Pohang Steel Company (POSCO), Dongchon 5, Pohang, Kyungbuk 790-784, South Korea

**Keywords:** Nano-bullet, CO_2_, Supersonic nozzle, Gas-phase nucleation, Cleaning efficiency

## Abstract

Removal of nanometer-sized contaminant particles (CPs) from substrates is essential in successful fabrication of nanoscale devices. The particle beam technique that uses nanometer-sized bullet particles (BPs) moving at supersonic velocity was improved by operating it at room temperature to achieve higher velocity and size uniformity of BPs and was successfully used to remove CPs as small as 10 nm. CO_2 _BPs were generated by gas-phase nucleation and growth in a supersonic nozzle; appropriate size and velocity of the BPs were obtained by optimizing the nozzle contours and CO_2_/He mixture fraction. Cleaning efficiency greater than 95% was attained. BP velocity was the most important parameter affecting removal of CPs in the 10-nm size range. Compared to cryogenic Ar or N_2 _particles, CO_2 _BPs were more uniform in size and had higher velocity and, therefore, cleaned CPs more effectively.

## Background

The manufacturing yield of submicron-scale devices can be reduced by particulate contamination of their surfaces. Sizes of semiconductor device features are expected to decrease continuously, reaching 25 nm by 2015 for DRAM/flash memory devices, and together with this decrease, critical killer particle size is expected to decrease to 12.5 nm [1]. In the nanometer range, the adhesion force per contact area between a particle and surface increases linearly as particle size decreases, but the fluid drag force changes in proportion to the cross-sectional area of a particle [2] so the use of drag force to remove contaminant particles (CPs) becomes less efficient as CP size decreases [3-6].

One promising method of removing very small CPs is the cryogenic aerosol technique in which the contaminated surface is bombarded by fine, high-velocity bullet particles (BPs) made of volatile material. CPs on the surface can be removed if the energy transferred from BPs to the CP is sufficient to overcome the adhesion energy between the CP and the substrate [7,8]. In the conventional form of this technology, condensable gas is pre-cooled close to liquid nitrogen temperature and, then, expanded through a simple nozzle such as a cylindrical hole. Cooling liquefies some of the condensable gas; expansion of the gas through the nozzle atomizes the liquid into fine droplets, and adiabatic cooling causes solid particles to form during further expansion. Typical particle size is a few microns, and typical velocity is 100 m/s. The minimum size of CP that can be removed efficiently using these micron-sized particles is approximately 50 nm [6-10].

Simulations suggest that reducing the size of the BP and projecting it at higher velocity can increase the effectiveness of CP removal even if the kinetic energy of the BPs is not changed [11]. If the BP is too big compared to the target CP, the atoms or molecules released by the BP when it fragments after collision with the CPs or the substrate may prevent the CP from leaving the surface [11]; this implies that BPs must be less than some maximum size if they are to effectively remove nanometer-size CPs. A new particle beam technique using Ar BPs has been developed and used to remove CPs of 20-nm size from flat surfaces and trenches [12,13]; its success was attributed to the nanometer size and supersonic velocity of the BPs.

Despite the success, two practical difficulties using Ar BPs remain: (1) BP size is very sensitive to temperature [14], so it must be controlled very accurately to achieve size uniformity, and (2) BP velocity is limited by low temperature. Generating BPs at room temperature (RT) would eliminate the need to control temperature and increase BP velocity. The resulting uniform, fast BPs should improve removal of nanometer CPs.

The aim of this study is, thus, to explore the possibility of extending the capability of the nanoparticle beam technique down to 10-nm size by increasing BP velocity. Adhesion force per contact area for a 10-nm particle is twice as high as that for a 20-nm particle, so an appropriate combination of size and velocity of the BPs is needed to overcome this high adhesion force. Pure CO_2 _or a mixture of CO_2 _and He was used to generate nano-sized CO_2 _BPs with supersonic velocity. This technique is unique in that nano-sized CO_2 _BPs are used and that 10-nm CPs are removed.

## Methods

**Figure 1:**
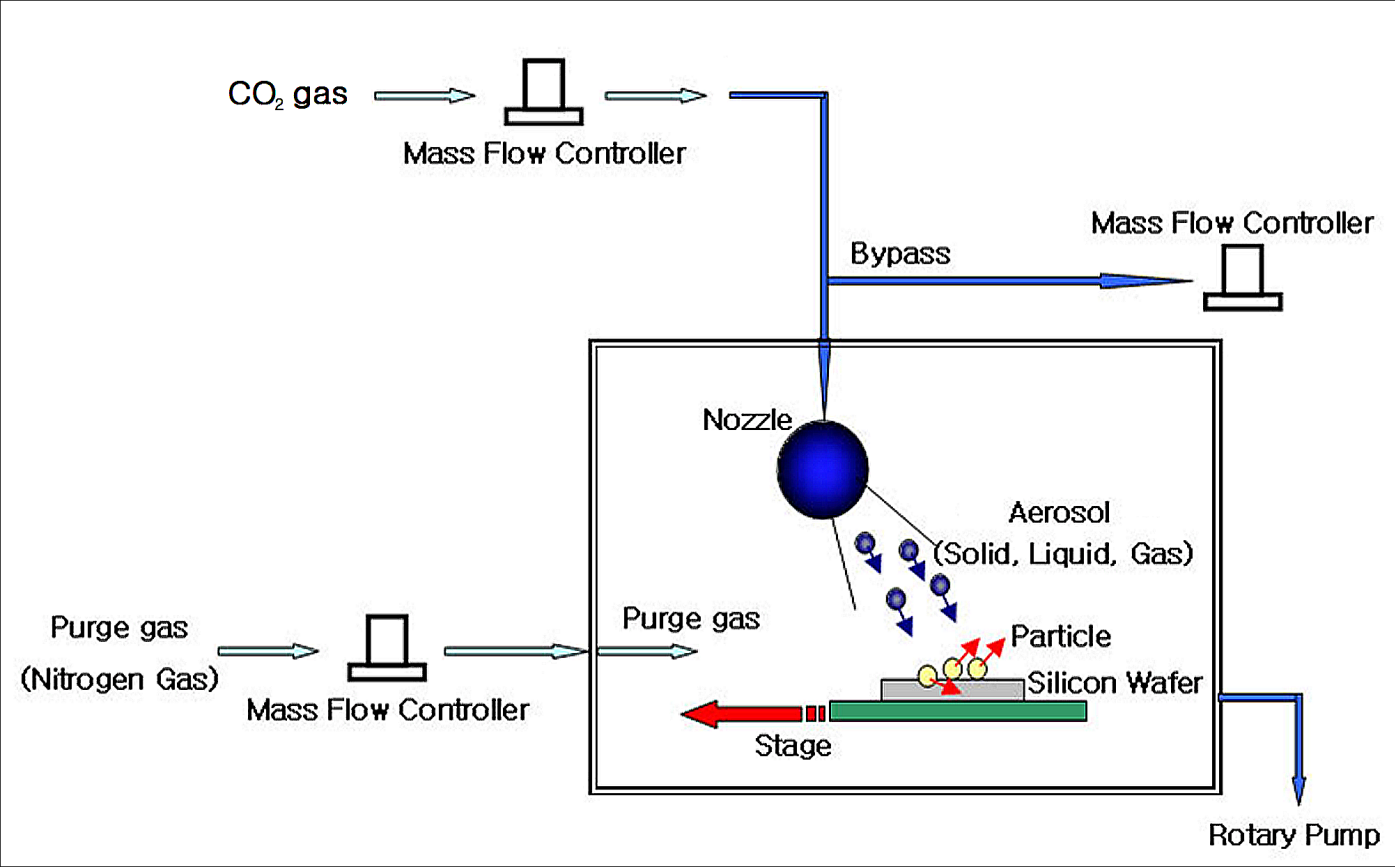
**Schematic of the experimental setup**.

A condensable gas or gas mixture at RT was expanded directly to a vacuum through a supersonic de Laval nozzle (Figure 1). During supersonic expansion through the nozzle, tiny condensation nuclei form and grow; their final size can be controlled by adjusting the stagnation pressure, the back pressure of the vacuum chamber, and the nozzle geometry. BPs are formed entirely by homogeneous nucleation and growth downstream of the nozzle throat.

To generate BPs traveling at the greatest possible supersonic velocity, pure CO_2 _or CO_2_/He mixture gas was used. Stagnation pressure was varied in the range of 5-50 bar; stagnation temperature was kept at RT. The shape and size of the nozzle were adjusted to make the Mach number at the nozzle exit > 5.0 over the whole range of experimental conditions. The nozzle is nearly conical at the throat but rapidly expands near the exit to compensate for boundary layer growth and to prevent shock wave formation.

In all trials, the CPs were 10-nm Al_2_O_3 _particles (Aldrich No. 642991, Sigma-Aldrich Corporation, St. Louis, MO, USA); they were generated by spin coating a drop of Al_2_O_3 _solution onto a wafer which was then dried for a few days in dry N_2 _gas. Based on the nucleation rate predicted by a classical nucleation theory and measured particle velocity, the rate of bombardment by the BPs is estimated at about 1,000-10,000/s on every square of 20-nm side [15]. Thus, cleaning was completed within 1-2 s of exposure in all the cases shown. The cleaning was unprecedentedly fast which is attributed to the extremely high rate of nuclei formation during homogeneous nucleation under highly supersaturated conditions. Evaporation of CO_2 _BPs after collision on the wafer surface generates a gas flow of high velocity which is strong enough to carry the dislodged CPs to the vent exit.

Scanning electron microscope (SEM) images were taken before and after cleaning. The cleaning efficiency was determined by the change in the surface area covered by the CPs, and the removal area was measured using the Image J, a free software distributed by the US National Institute of Health (MD, USA). Nozzle-wafer distance and the angle of the particle beam incident on the wafer varied, but the photographs shown were all taken when the incident angle of the particle beam was 30° to the normal. Chamber pressure was maintained low enough to ensure supersonic flow inside the nozzle.

## Results and discussion

**Figure 2:**
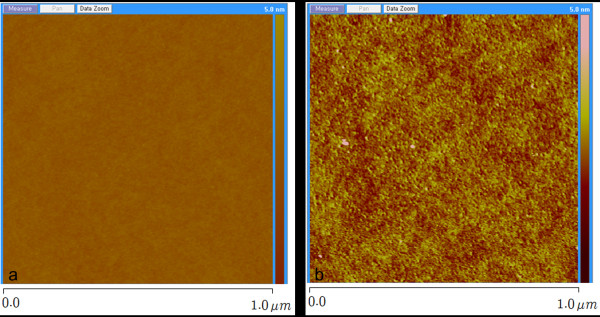
**AFM images**. Before (**a**) and after (**b**) images of dents on a photo resist film made by BPs generated by nucleation and growth with pure CO_2 _at 30 bar.

**Figure 3:**
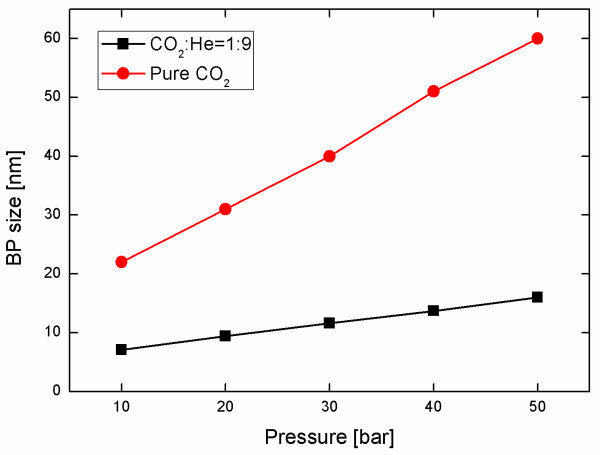
**Variation of BP size with generation pressure**.

First, the size of the generated CO_2 _BPs was determined using atomic force microscope (AFM) to measure dents formed in a photo resist (PR) film on a wafer when bombarded by the BPs. The hardness of the PR film was controlled so that the BPs made dents of size comparable to the BP size [7,8]. In previous studies, BPs generated by liquid atomization of pure Ar at 4,000 Torr (5.33 bar) formed dents of 1-10-μm diameter [7,8,12]. Instead, the BPs generated in this study by homogeneous nucleation and growth at 30 bar of pure CO_2 _gas formed dents of only 5-50 nm (Figure 2). Average size of the BPs changed almost linearly with stagnation gas pressure, and BPs made with 1:9 CO_2_/He mixture were about 1/3 size of the BPs made with pure CO_2 _gas at the same total pressure (Figure 3). Compared to previous studies based on atomization techniques [7,8,12], BPs generated in this study were much smaller and had a narrower size distribution. The size of the BPs used in this study is on the same order of magnitude as that of the CPs. Refer to the study of Bae et al. [14] for particle size estimation and the works of Lee et al. [12] and Hwang et al. [13] for experimental details.

**Figure 4:**
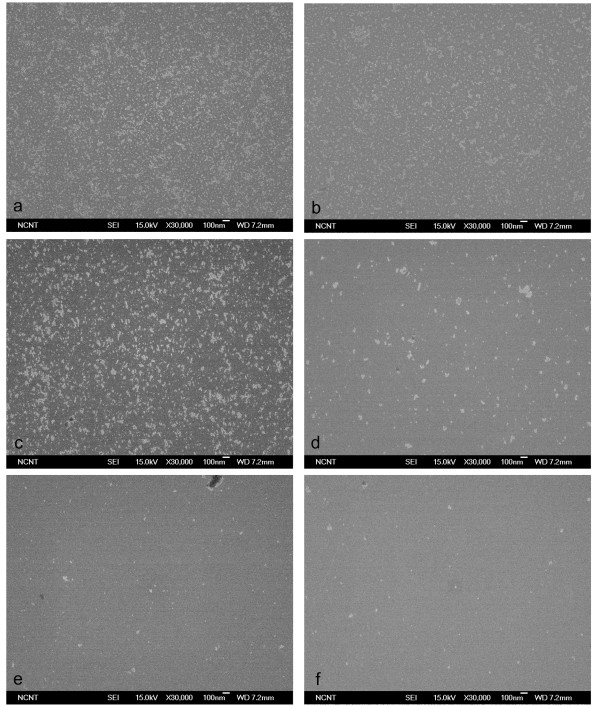
**SEM images**. SEM images before (**a**) and after (**b**-**f**) cleaning of 10-nm Al_2_O_3 _particles on Si surface using pure CO_2 _BPs generated with original throat diameter.

BP size affected the effectiveness with which CPs were cleaned from a flat Si wafer (Figure 4a). The mean size of the BPs generated by homogeneous nucleation and growth in a supersonic nozzle increase almost linearly with pressure [14], so BPs of different sizes were generated using pure CO_2 _gas at different pressures of 10-50 bar. No CPs were removed at 10-20 bar (Figure 4b, c), and partial removal was observed at 30 bar (Figure 4d). Substantial removal was attained at 50 bar (Figure 4f), but the use of higher pressures did not give any further improvement. These results imply that cleaning effectiveness increases with BP size only up to a certain BP size.

**Figure 5:**
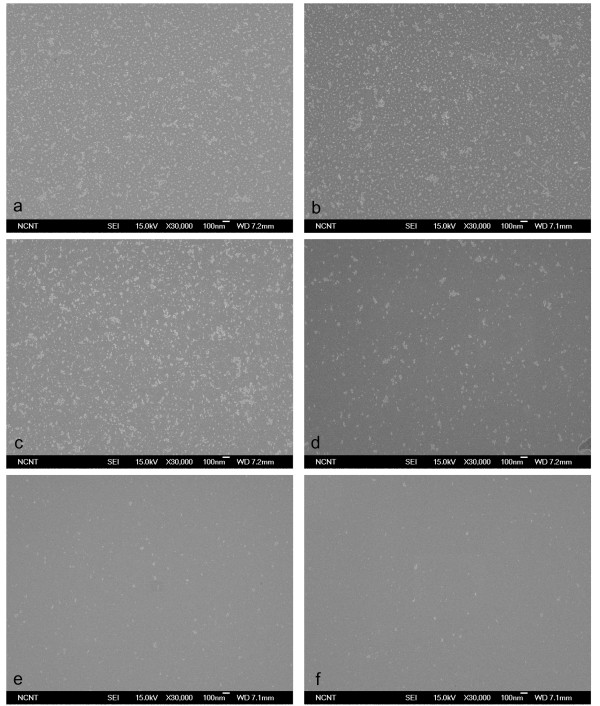
**SEM images**. SEM images before (**a**) and after (**b-f**) cleaning of 10-nm Al_2_O_3 _particles on Si surface using pure CO_2 _BPs generated with 1/3 throat diameter.

**Figure 6:**
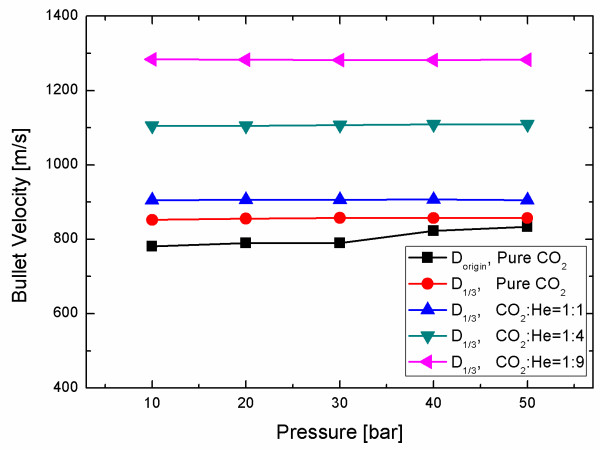
**Flow velocity at the nozzle exit at different pressures and gas compositions **. D, diameter.

BP velocity also affects cleaning effectiveness. Because absolute flow speed increases only slightly with pressure in high Mach number conditions [14], nozzles with the same contours but different throat diameters were used to attempt to increase BP velocity, based on the theoretical reasoning that a smaller throat gives a higher Mach number and velocity at the nozzle exit [16]. However, the reduction of the nozzle throat diameter over a factor of 3 did not give any appreciable improvement in CP removal (Figure 5). Measured at the nozzle exit using a Pitot micro-tube, flow speed or BP velocity increased by as much as 7% during pressure excursions from 10 to 50 bar, but the reduced throat gave an additional increase of only 2% (Figure 6). The negligible effect of the nozzle throat on the flow velocity at the exit can be attributed to the thick boundary layer occupying a substantial fraction of the nozzle cross-section in a micro-nozzle: the size of the real or hydrodynamic throat formed downstream of the geometric throat change only slightly even when the diameter of the geometric nozzle throat changes substantially.

Therefore, a CO_2_/He mixture gas was used to increase BP velocity. Because supersonic flow speed varies with specific-heat ratio (R) and molecular weight (M) of the gas as approximately (R/M)^1/2^, the use of light carrier gas with high R like He is expected to give very high BP velocity. According to inviscid flow theory, expected velocity increase over pure CO_2 _is approximately 45% for a 1:1 CO_2_/He mixture, 120% for a 1:4 mixture and 180% for a 1:9 mixture, but measured increase in velocity with mixture fraction was much smaller than expected, i.e., 5%, 30%, and 50% for 1:1, 1:4, and 1:9 CO_2_/He mixtures, respectively (Figure 6); this difference can be attributed again to the increased boundary layer thickness due to the highly viscous He gas.

**Figure 7:**
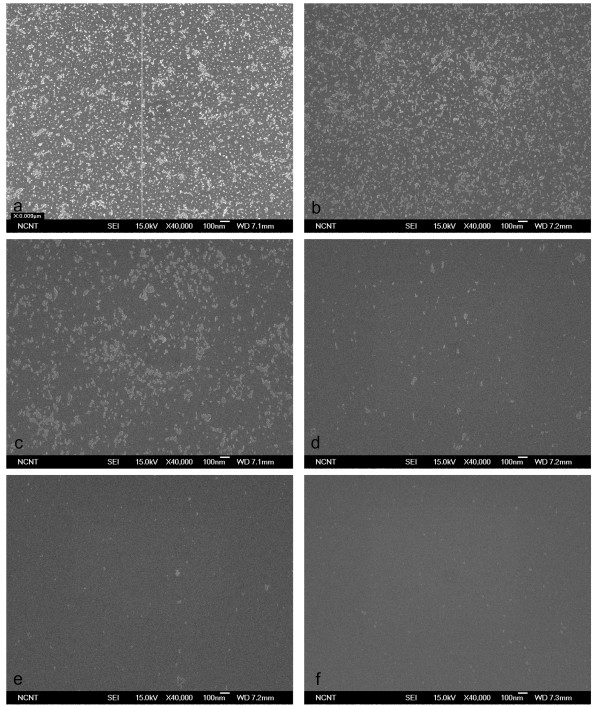
**SEM images of 10-nm Al_2_O_3 _particles before and after cleaning using 1:1 CO_2_/He BPs**. SEM images before (**a**) and after (**b-f**) cleaning of 10-nm Al_2_O_3 _particles on Si surface using 1:1 CO_2_/He BPs generated with 1/3 throat diameter.

**Figure 8:**
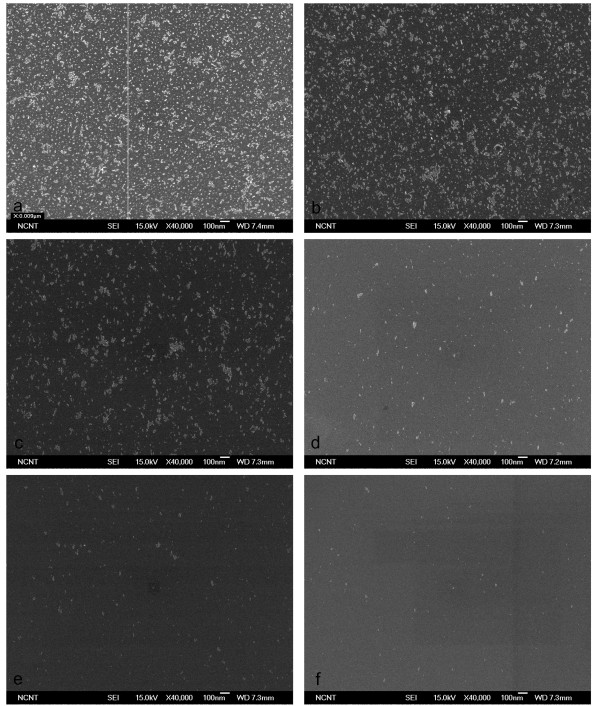
**SEM images of 10-nm Al_2_O_3 _particles before and after cleaning using 1:4 CO_2_/He BPs**. SEM images before (**a**) and after (**b-f**) cleaning of 10-nm Al_2_O_3 _particles on Si surface using 1:4 CO_2_/He BPs generated with 1/3 throat diameter.

**Figure 9:**
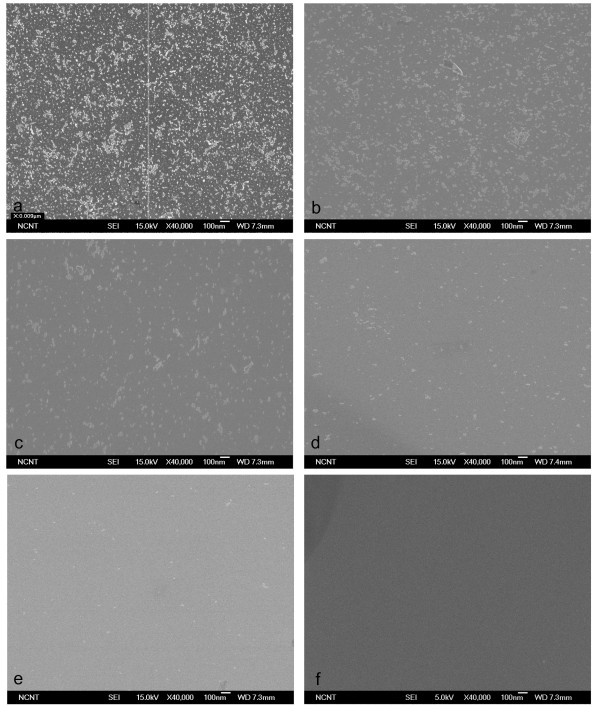
**SEM images of 10-nm Al_2_O_3 _particles before and after cleaning using 1:9 CO_2_/He BPs**. SEM images before (**a**) and after (**b-f**) cleaning of 10-nm Al_2_O_3 _particles on Si surface using 1:9 CO_2_/He BPs generated with 1/3 throat diameter.

**Figure 10:**
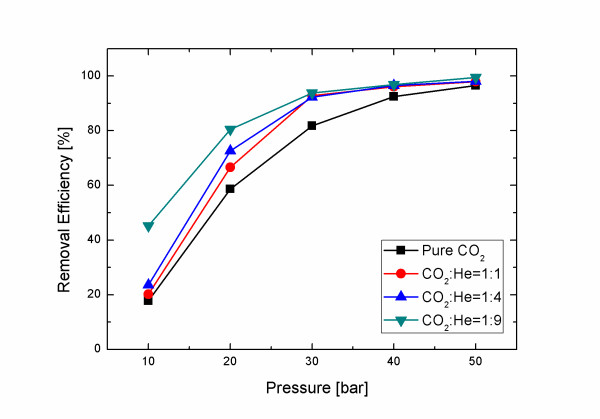
**Removal efficiency of 10-nm Al_2_O_3 _particles on Si surface with 1/3 throat diameter**.

Cleaning effectiveness increased with the proportion of He in the CO_2_/He mixture and with pressure. The 1:1 CO_2_/He mixture did not remove any CP from the wafer at pressures below 10 bar (Figure 7a, b); when pressure was increased to 20, 30, and 40 bar, most 20-nm CPs were removed (Figure 7c, d, e) but some 10-nm CPs remained (Figure 7f). The 1:4 CO_2_/He mixture cleaned the wafer slightly better than when using pure CO_2 _but cleaned almost the same as with 1:1 CO_2_/He at the same pressure (Figure 8a, b, c, d, e, f). The 1:9 CO_2_/He mixture began removing CP at 20 bar and removed 10-nm CPs almost completely at 50 bar (Figure 9). Change of removal efficiency with four different gas mixtures at five different pressures was summarized in Figure 10.

**Figure 11:**
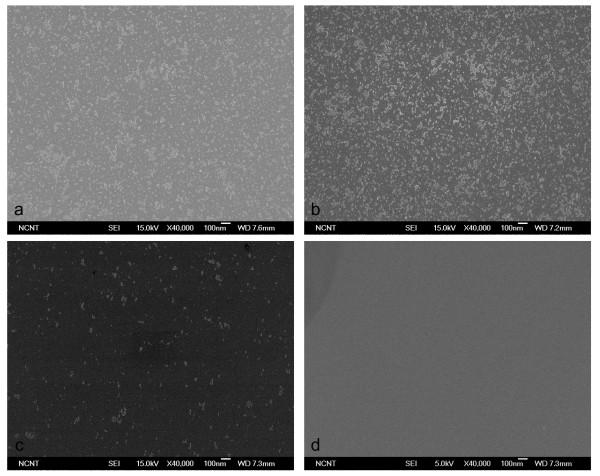
**SEM images after cleaning of 10-nm Al_2_O_3 _p articles using different ratios of CO_2_/He mixtures**. SEM images after cleaning of 10-nm Al_2_O_3 _particles on Si surface using (**a**) pure CO_2 _at 5 bar, (**b**) 1:1 CO_2_/He at 10 bar, (**c**) 1:4 CO_2_/He at 25 bar, and (**d**) 1:9 CO_2_/He at 50 bar with 1/3 throat diameter.

Because BP size is almost linearly proportional to the partial pressure of the condensable species, the similar level of cleaning among different gas mixtures implies that the effect of increased BP velocity is almost equal to the effect of the reduced BP size. When the removal performance was compared between BPs of almost the same size but generated using different gas mixtures with a CO_2 _partial pressure of 5 bar, only the 1:9 CO_2_/He mixture removed > 95% for 10-nm CPs (Figure 11).

The consistent increase in removal efficiency with He fraction at the same BP size implies that BP velocity has the greatest effect on the removal of nanometer-sized CPs. BP size is important only in that the BP must be big enough to have total kinetic energy sufficiently higher than the CP-surface adhesion energy, but at the same time smaller than a critical size at which dispersal of removed CPs is inhibited by the atoms or molecules emitted during breakup of the BP. If the useful BP size is on the same order as the target particle as was observed in this study, using BPs that are smaller than the conventional micron particles by a factor of about 100 is expected to damage the substrate much less than micron particles because damage is proportional to the total kinetic energy of each BP.

**Figure 12:**
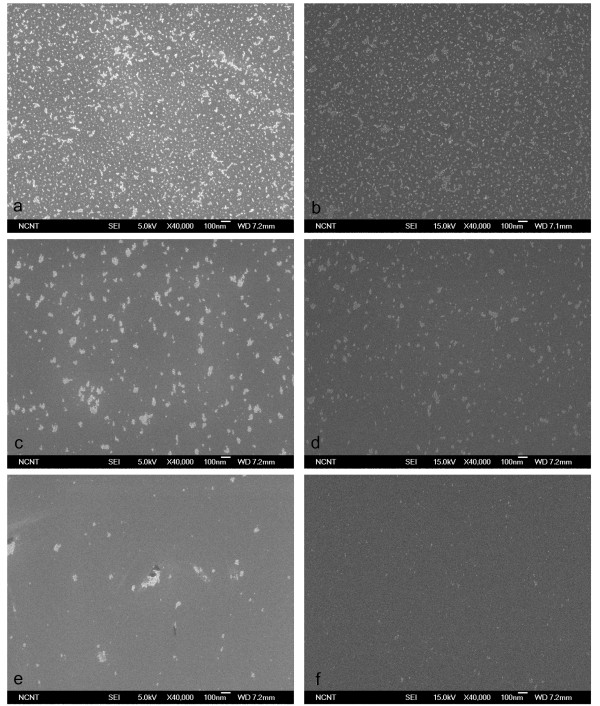
**SEM images after cleaning of 10-nm Al_2_O_3 _particles at different chamber pressures**. SEM images after cleaning of 10-nm Al_2_O_3 _particles on Si surface using pure CO_2 _at different chamber pressures (P_c_): (**a**) P_c _= 3.7 Torr at 10 bar, (**b**) P_c _= 0.37 Torr at 10 bar, (**c**) P_c _= 8.4 Torr at 30 bar, (**d**) P_c _= 0.84 Torr at 30 bar, (**e**) P_c _= 11 Torr at 50 bar, and (**f**) P_c _= 1.1 Torr at 50 bar.

The cleaning performance is closely related with chamber pressure. To confirm the interrelation between cleaning performance and chamber pressure, cleaning was done at different background pressures. Cleaning effectiveness decreased with increase in chamber pressure (Figure 12); this decrease can be attributed to insufficient expansion or acceleration at high chamber pressure conditions.

## Conclusions

The feasibility of removing 10-nm CPs from a substrate using nano-sized volatile BPs was proven experimentally: (1) by controlling the nozzle contour and expansion pressure, CO_2 _particles of 5-50-nm size were generated by gas-phase nucleation and growth; (2) when using a carrier gas mixture of 1:9 CO_2_/He, the particle beam generated in this study could remove 10-nm CPs with efficiency > 95%; (3) the velocity of the BPs was the most important factor in determining the effectiveness of CP removal, and the use of light carrier gas (He) was effective in increasing flow velocity; (4) cleaning effectiveness increased with decrease in chamber pressure; and (5) compared to Ar or N_2 _particles generated by liquid atomization, CO_2 _BPs were more uniform in size and had higher velocity and, therefore, cleaned CPs more effectively.

## Abbreviations

AFM: Atomic force microscope; BP: Bullet particle; CP: Contaminant particle; M: Molecular weight; P_c_: Chamber pressure; PR: Photo resist; R: Specific-heat ratio; RT: Room temperature; SEM: Scanning electron microscope.

## Competing interests

The authors declare that they have no competing interests.

## Authors' contributions

IK carried out the cleaning experiment and analyzed the experimental results. JWL carried out the theoretical analysis of the cleaning process, determined the experimental conditions, and wrote the manuscript. KH designed the nozzle and measured the flow field near the nozzle. All authors read and approved the final manuscript.
